# Roscovitine strongly enhances the effect of olaparib on radiosensitivity for HPV neg. but not for HPV pos. HNSCC cell lines

**DOI:** 10.18632/oncotarget.22005

**Published:** 2017-10-24

**Authors:** Frank Ziemann, Steve Seltzsam, Kristin Dreffke, Stefanie Preising, Andrea Arenz, Florentine S.B. Subtil, Thorsten Rieckmann, Rita Engenhart-Cabillic, Ekkehard Dikomey, Andrea Wittig

**Affiliations:** ^1^ Department of Radiotherapy and Radiooncology, Philipps-University Marburg, University Hospital GieΔen and Marburg, Marburg, Germany; ^2^ Laboratory for Radiobiology & Experimental Radiooncology, University Medical Center Hamburg Eppendorf, Hamburg, Germany; ^3^ Department of Otolaryngology and Head and Neck Surgery, University Medical Center Hamburg Eppendorf, Hamburg, Germany

**Keywords:** human papillomavirus, roscovitine, homologous recombination, ionizing radiation, HNSCC

## Abstract

At present, advanced stage human Papillomavirus (HPV) negative and positive head and neck squamous cell carcinoma (HNSCC) are treated by intense multimodal therapy that includes radiochemotherapy, which are associated with relevant side effects. Patients with HPV positive tumors possess a far better prognosis than those with HPV negative cancers. Therefore, new therapeutic strategies are needed to improve the outcome especially of the latter one as well as quality of life for all HNSCC patients. Here we tested whether roscovitine, an inhibitor of cyclin-dependent kinases (CDKs), which hereby also blocks homologous recombination (HR), can be used to enhance the radiation sensitivity of HNSCC cell lines.

In all five HPV negative and HPV positive cell lines tested, roscovitine caused inhibition of CDK1 and 2. Surprisingly, all HPV positive cell lines were found to be defective in HR. In contrast, HPV negative strains demonstrated efficient HR, which was completely suppressed by roscovitine. In line with this, for HPV negative but not for HPV positive cell lines, treatment with roscovitine resulted in a pronounced enhancement of the radiation-induced G2 arrest as well as a significant increase in radiosensitivity. Due to a defect in HR, all HPV positive cell lines were efficiently radiosensitized by the PARP-1 inhibitor olaparib. In contrast, in HPV negative cell lines a significant radiosensitization by olaparib was only achieved when combined with roscovitine.

## INTRODUCTION

Two main etiologies of head and neck squamous cell carcinoma (HNSCC) are recognized that reveal distinct biological characteristics. One is caused by infection with human Papillomavirus (HPV pos.), the other is mainly driven by noxa, such as tobacco and alcohol (HPV neg.) [1]. In total, worldwide incidence rates for HNSCC remain high, but a huge variation for different regions is noticed and trends mostly depend on smoking and sexual behavior. In the western world the incidence of HPV pos. HNSCC appears to rise, while the incidence of HPV neg. HNSCC stabilized or even slightly decreases in some countries [2].

Today, both HNSCC entities are treated by a simultaneous cisplatin-based radiochemotherapy as a definitive concept or in the adjuvant setting after radical surgery, if tumors are in an advanced stage [1]. For HPV pos. HNSCC this treatment results in a very good outcome with an eight year overall survival reaching 80 %. In contrast, for HPV neg. HNSCC, survival rates are still unfavorable with 30 % overall survival after the same follow-up period [3]. For both entities, treatment is frequently associated with severe side effects [3]. Therefore, there is an urgent need to develop new therapeutic strategies, (1) to improve the outcome especially for HPV neg. HNSCC and (2) to increase the quality of live for patients suffering of HNSCC in general.

Many different approaches were initiated already to implement new therapeutic strategies that specifically target HNSCC [4]. However, even the most promising concept, the inhibition of the epidermal growth factor receptor (EGFR) by cetuximab failed to improve survival rates when combined with cisplatin-based radiochemotherapy [5].

In order to achieve a tumor-specific effect, new treatments have to exploit tumor-specific defects, allowing for an individualized therapy. In this context cell-cycle regulation, which is accomplished by a complex network of different cyclin-dependent kinases (CDK) and respective cyclins, is considered to be of great relevance, because tumors often show defects in this process [6]. As a consequence, inhibition of specific CDKs is regarded as a promising tool to establish a tumor-specific therapy [7].

Roscovitine was one of the first CDK inhibitors targeting mainly CDK1 and CDK2 [8]. The cytotoxic and anti-proliferative activity of this drug has already been shown in many different types of cancer both *in vitro* and *in vivo* [9, 10] including HPV neg. HNSCC cell lines [11]. A recent report indicated that roscovitine is especially effective in HPV pos. HNSCC cell lines suggesting that treatment with roscovitine may represent a selective and safe targeted therapeutic option against HPV pos. HNSCC [12]. In contrast, the results obtained in clinical trials with roscovitine as monotherapy were mostly disappointing [8]. Two phase I trials reported stabilization of the disease or moderate tumor response in advanced stage solid tumors [13, 14].

Besides its activity as single agent, roscovitine *in vitro* can modulate the response of classical chemotherapeutics [15] as well as ionizing radiation [16]. An increase in cellular radiosensitivity was observed *in vitro* for breast cancer [16], nasopharyngeal carcinoma [9], as well as non-small cell lung carcinoma [17, 18]. In all studies, this effect was primarily attributed to an enhanced apoptosis as well as a depressed repair of DNA double strand-breaks (DSB).

We now tested, whether roscovitine may also be used to enhance the radiosensitivity of HPV neg. and pos. HNSCC cell lines. It is well known that the cellular radiosensitivity is primarily determined by the DSB repair efficiency [19]. Roscovitine can specifically inhibit homologous recombination (HR) [20], which is one of the two main DSB repair pathways active in mammalian cells [7]. An inhibition of CDKs as can be achieved by roscovitine, which blocks the activation of DNA repair proteins, such as Exo1, BRCA1 and CtIp and thereby suppresses the initiation of HR prior to RAD51 foci formation [21–23]. Thus, the concept of this project was to target DSB repair, especially HR, through roscovitine to achieve a relevant radiosensitization.

It was previously shown by us and others, that HPV pos. cell lines on average possess a higher radiosensitivity than HPV neg. cell strains [24–26]. This sensitivity was associated with an elevated and prolonged radiation-induced G2 arrest as well as a defective DSB repair [24, 25].

We describe here that roscovitine can be used to down-regulate HR in HPV neg. cell lines. Whereas roscovitine did not affect HPV pos. cells because HR was already impaired in these cell lines. In line with this, an increase in radiosensitivity by roscovitine was only achieved for HPV neg. but not for HPV pos. cell lines. The radiosensitivity of HPV neg. cells was further enhanced, when roscovitine was combined with the PARP1 inhibitor olaparib. In contrast, radiosensitivity of HPV pos. cell lines was only enhanced when pretreated with olaparib alone. The changes in radiosensitivity were strongly correlated with the respective variation in DSB repair capacity. These data confirm that the inhibition of DSB repair is an optimal tool to enhance the cellular radiosensitivity for both HPV neg. and pos. HNSCC cell lines. However, we also show that different strategies of radiosensitization are necessary to address the special characteristics of HPV neg. and pos. cell lines, respectively.

## RESULTS

### Roscovitine affects proliferation as well as CDK1 and CDK2 activity in both HPV neg. and HPV pos. cell lines

**Figure 1:**
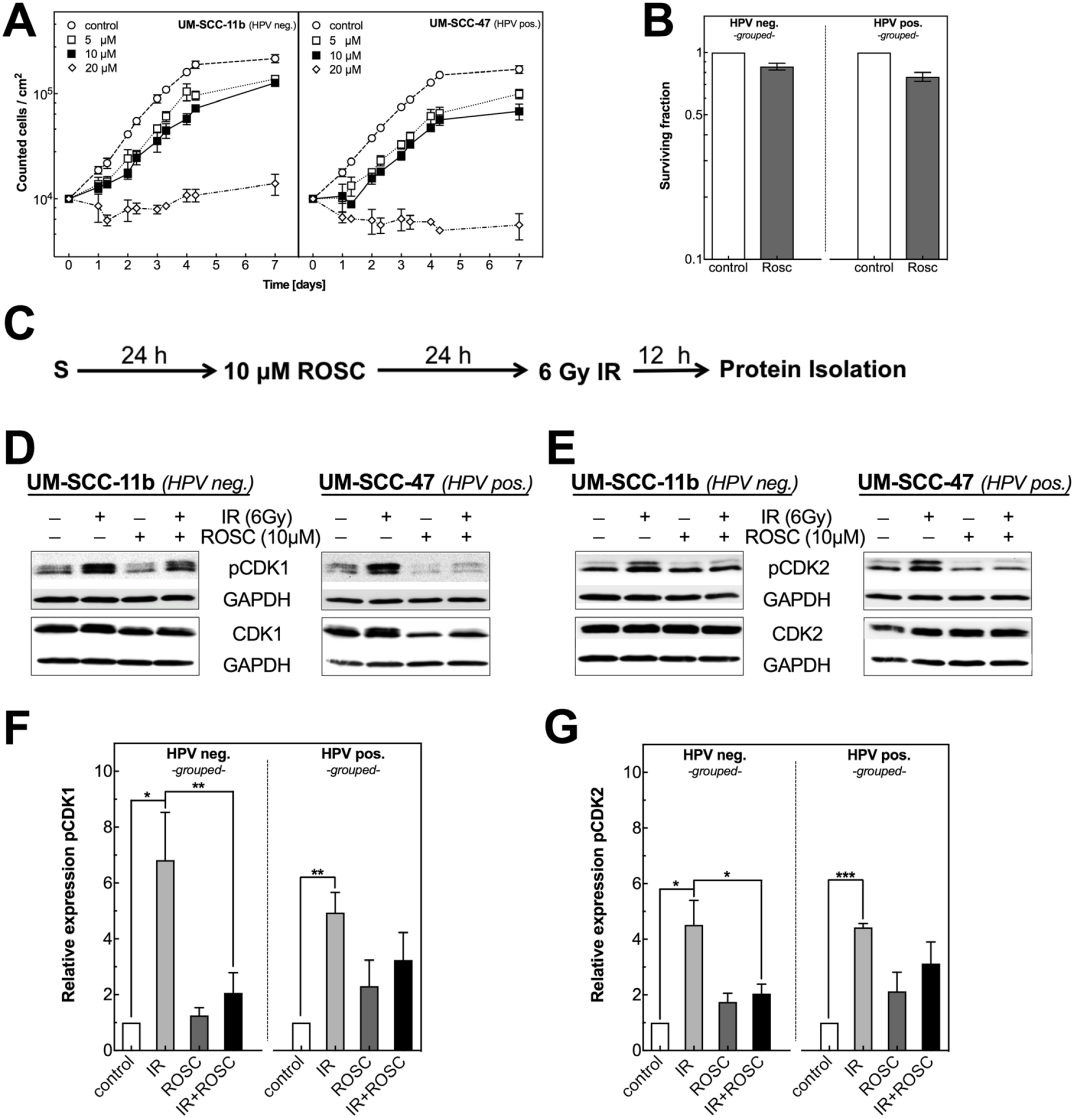
Roscovitine affects proliferation as well as CDK1, CDK2 phosphorylation in HPV neg. and pos. HNSCC cell lines **(A)** Growth curves of UM-SCC-11b (HPV neg., left panel) and UM-SCC-47 (HPV pos., right panel) incubated with roscovitine (0-20 μM). Cell numbers were measured for up to 7 days. **(B)** Clonogenic survival of HPV neg. (left panel) and HPV pos. (right panel) cell lines measured after incubation with DMSO or roscovitine (10 μM) for 48 h. Colony formation was measured for all ten cell lines and then grouped according to the HPV status. **(C)** Scheme for Protein isolation after ionizing radiation (IR) and roscovitine (ROSC) treatment. **(D/E)** Representative western blots showing the expression of CDK1 and pCDK1 as well as CDK2 and pCDK2 for one HPV neg. (left panels) and one HPV pos. (right panels) cell line. Cells preincubated with ROSC (10 μM) for 24 h were irradiated with 6 Gy followed by an incubation for 12 h before extracting proteins. GAPDH served as loading control. **(F/G)** Quantification of pCDK1 (F) and pCDK2 (G) in five HPV neg. (left panels) and five HPV pos. (right panels) cell lines after treatment with ROSC and/or IR. Bars represent grouped mean values ± SEM of 5 HPV neg. / 5 HPV pos. cell lines with at least 3 independent experiments performed per cell line; S = seeding ; ^*^p<0.05, ^**^p<0.01, ^***^p<0.001.

CDKs are known for their essential role in cell-cycle regulation and proliferation [6]. Therefore, we first tested in how far proliferation as well as pCDK1 and 2 are affected by the CDK inhibitor roscovitine. At low concentrations of 5 and 10 μM only a moderate delay in proliferation was seen, in contrast to a strong delay (HPV neg.) or even complete block (HPV pos.) obtained at 20 μM (Figure 1A). With increasing concentration of roscovitine also more trypan blue positive cells, indicating dead cells, were counted (data not shown).

Figure 1B shows the effect of an incubation with 10 μM roscovitine for 48 h on the clonogenic survival as determined for all five HPV neg. and five HPV pos. cell lines (Supplementary Figure 4 for individual cell lines). The surviving fraction (SF) was moderately reduced in all HNSCC cell strains. This effect was slightly but non-significantly stronger for HPV pos. cell lines when compared to HPV neg. cell strains (Figure 1B, p=0.11). HPV pos. cell lines also showed a slightly higher level of apoptosis when treated by roscovitine (Supplementary Figure 6).

Next, we tested the effect of ionizing radiation and roscovitine on the activity of CDK1 and CDK2, which is considered to be its primary target [8]. The CDK activity is generally regulated by a cell-cycle phase dependent expression of corresponding cyclins causing a de-phosphorylation and phosphorylation at specific sites. For activation, CDK1 and 2 have to be de-phosphorylated at T14 and Y15 and phosphorylated at T161 in case of CDK1 and T160 for CDK2. The de-phosphorylation as final step is executed by Cdc25 in late S and G2 phase [27]. When cells are exposed to ionizing radiation the de-phosphorylation is inhibited by checkpoint kinases Chk1/2, which block Cdc25 leading to an increase of triple phosphorylated, inactivated CDK1 and CDK2, finally causing a G2 block [28].

Figure 1D–1G shows the effect of roscovitine on the phosphorylation of CDK1 at T161 and for CDK2 at T160 alone as well as when combined with irradiation (Supplementary Figure 1 for individual plots). Roscovitine alone was found to cause a small increase of pCDK1 and pCDK2, which appeared to be slightly stronger for HPV pos. cell lines. Compared to that a much stronger increase of phosphorylation was seen after exposure to 6 Gy alone, whereby the relative increase (RI) in pCDK1 and pCDK2 were fairly similar for HPV neg. and pos. cell lines. When pretreated by roscovitine, the radiation-induced increase of both pCDK1 and 2 was clearly reduced especially for HPV neg. cell lines (Figure 1F and 1G, dark bars). These data demonstrate that 10 μM roscovitine clearly impairs the radiation-induced activation of both CDK1 and 2.

### Roscovitine represses RAD51 foci formation only in HPV neg. cell lines

**Figure 2:**
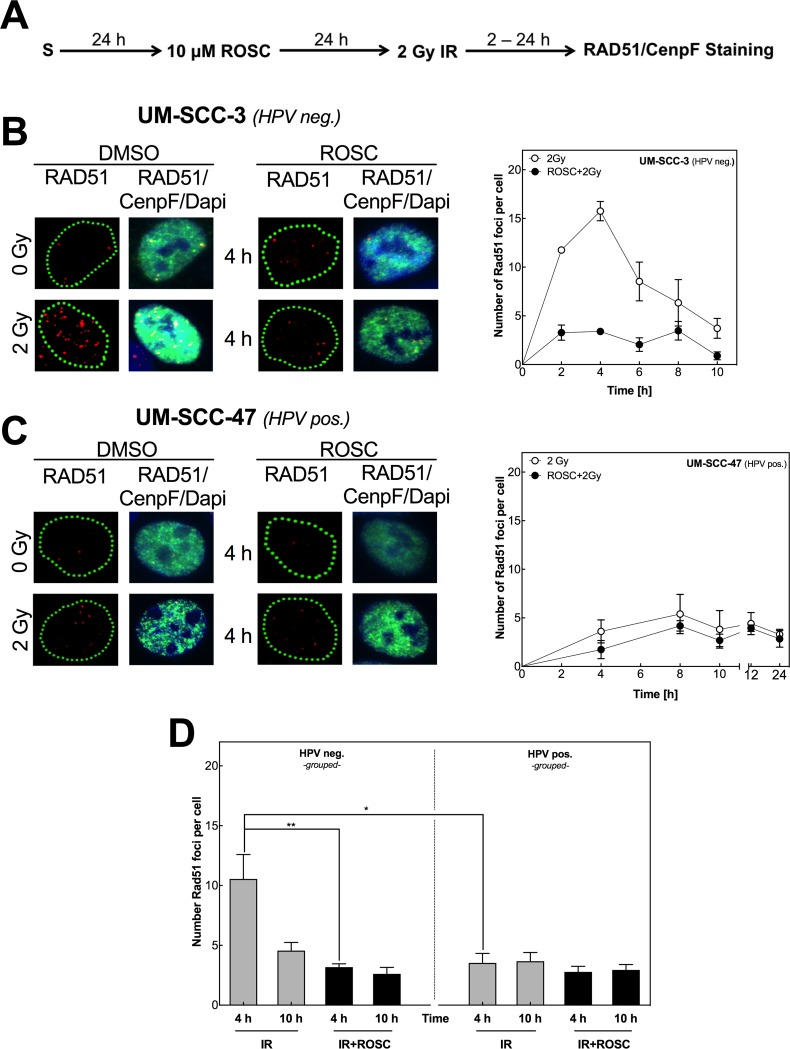
Roscovitine inhibits RAD51 foci formation only in HPV neg. cell lines **(A)** Scheme of RAD51 staining after ionizing radiation (IR) and roscovitine (ROSC) treatment. **(B/C)** Representative pictures of RAD51 foci (red) in G2-phase cells (CenpF pos., green) after IR with a dose of 2 Gy with or without ROSC pretreatment (10 μM, 24 h) in one HPV neg. (UM-SCC-3, A, left side) and one HPV pos. (UM-SCC-47, B, left side) cell line. Quantification of RAD51 foci in CenpF pos. cells after IR with 2 Gy at indicated time points. **(D)** Quantification of RAD51 foci in all five HPV neg. (left panel) and all five HPV pos. (right panel) cells 4 and 10 h after 2 Gy IR ± ROSC preincubation. Bars represent grouped mean values ± SEM of 5 HPV neg. / 5 HPV pos. cell lines with 3 independent experiments performed per cell line; S = seeding; ^*^p<0.05, ^**^p<0.01; Quantification was performed by counting at least 100 nuclei per sample.

Besides their role in cell-cycle regulation, CDK1 and CDK2 are known to be relevant for the repair of DNA DSBs by HR [29]. The quantification of RAD51 foci in cells stained positive for the centromeric protein F (CenpF+) is a well-established method to study HR in cells in G2 phase [30]. Measured with this method, in the HPV neg. cell line UM-SCC-3 the number of RAD51 foci increased with time after exposure to irradiation with 2 Gy with a peak after 4 h followed by a decline up to 10 h (Figure 2B, right chart). This increase was almost completely suppressed, when UM-SCC-3 cells were pretreated with roscovitine. Surprisingly, in the HPV pos. cell line UM-SCC-47, irradiation only resulted in a minor induction of RAD51 foci and – as a consequence – a small reduction only was seen when pretreated with roscovitine (Figure 2C, right chart).

To confirm whether this is a general phenomenon in HNSCC cell lines, RAD51-foci formation was additionally measured in the other four HPV neg. and four pos. cell lines 4 and 10 h post IR (Figure 2D, Supplementary Figure 2). In line with data described above, we observed a clear induction of RAD51 foci for the other HPV neg. HNSCC cell lines 4 h post IR with a strong decline followed thereafter (Figure 2D, left chart). Exceptionally UT-SCC-33 cells showed only a moderate formation of RAD51 foci. In all HPV neg. cell lines this induction of RAD51 foci was almost completely abolished when pretreated with roscovitine (p=0.007). Surprisingly, in HPV pos. cell lines, RAD51-foci formation was strongly reduced when compared to HPV neg. cell lines with a somewhat moderate formation of foci only in UM-SCC-104 cells (Figure 2D; Supplementary Figure 2, right charts). The fact that RAD51-foci formation is reduced in HPV pos. cell lines when compared to HPV neg. cell lines strongly suggests that HPV pos. cell lines are characterized by an impaired HR. In contrast to the HPV neg. cell lines the addition of roscovitine had only little impact on RAD51-foci formation in HPV pos. cell strains, which further supports the finding of an intrinsic HR defect.

### Roscovitine strongly enhances the radiation-induced G2 arrest in HPV neg. but not in HPV pos. cell lines

**Figure 3:**
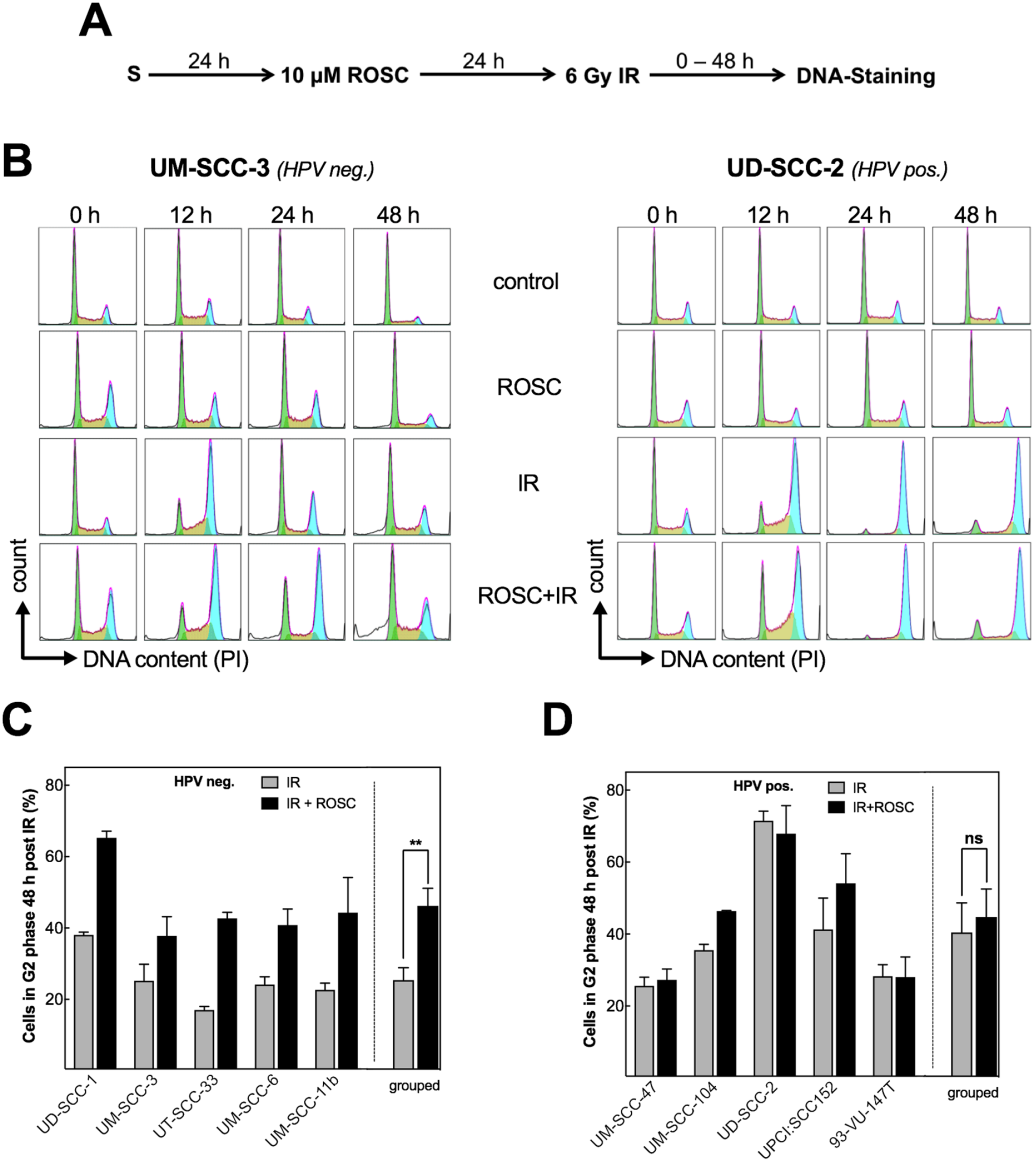
Roscovitine leads to additional G2 arrest only in HPV neg. cells **(A)** Scheme of cell-cycle analysis after ionizing radiation (IR) and roscovitine (ROSC) treatment. **(B)** Representative DNA histograms of UM-SCC-3 (HPV neg., left panel) and UD-SCC-2 (HPV pos., right panel) cells in control samples and 0 - 48 h after treatment with ROSC (10 μM) and/or IR (6 Gy); G1-phase cells: green, S-phase cells: orange, G2-phase cells: blue. **(C/D)** Quantification of the G2 phase fraction 48 h after IR ± roscovitine in HPV neg. (C) and HPV pos. (D) cell lines presented for the individual cell line and also grouped by HPV status. Mean values ± SEM with at least 3 independent experiments performed per cell line; S = seeding; ^**^p<0.01.

An impaired HR is generally known to result in a pronounced G2 arrest when cells have to deal with un-repaired DNA-damage [31]. Therefore, we tested whether the suppression of RAD51 foci formation by roscovitine is accompanied by a respective increase in G2 arrest. To this end, flow cytometry was used to measure the cell-cycle distribution at 0, 12, 24 and 48 h after treatment (Figure 3A and 3B, Supplementary Figure 3). Treatment of cells by 10 μM roscovitine alone results in a modest increase in G2 phase (Figure 3B, Supplementary Figure 3). A much stronger G2 arrest was seen after radiation with 6 Gy alone, which was especially pronounced in HPV pos. cell lines as previously already reported [24, 25]. Similar but less pronounced G2 arrest was seen after 2 Gy (data not shown). In HPV neg. cell lines the radiation-induced G2 arrest was strongly enhanced when cells were pretreated by roscovitine as can be especially seen 48 h after treatment (Figure 3B, left chart; Figure 3C, p=0.002). In contrast, in HPV pos. cell lines pretreatment by roscovitine did generally not alter the cell cycle distribution after irradiation (Figure 3B, right chart; Figure 3D). Solely for the HPV pos. cell line UM-SCC-47 a marked change was seen (Supplementary Figure 3). Here, roscovitine appears to prevent the radiation-induced G2 arrest by an increase in G1 suggesting the induction of a transient G1 arrest.

Overall these data demonstrate that roscovitine specifically enhances the radiation-induced G2 arrest in HPV neg. cell lines resulting in an amount similar to the one observed in HPV pos. cell lines after irradiation alone. Since 10 μM roscovitine alone did not affect cell cycle progression, the increase in G2 arrest can be considered to result from the strong inhibition of HR achieved by roscovitine in HPV neg. cell lines as described above.

### Roscovitine and olaparib affect DSB repair differently in HPV neg. and HPV pos. HNSCC cell lines

**Figure 4:**
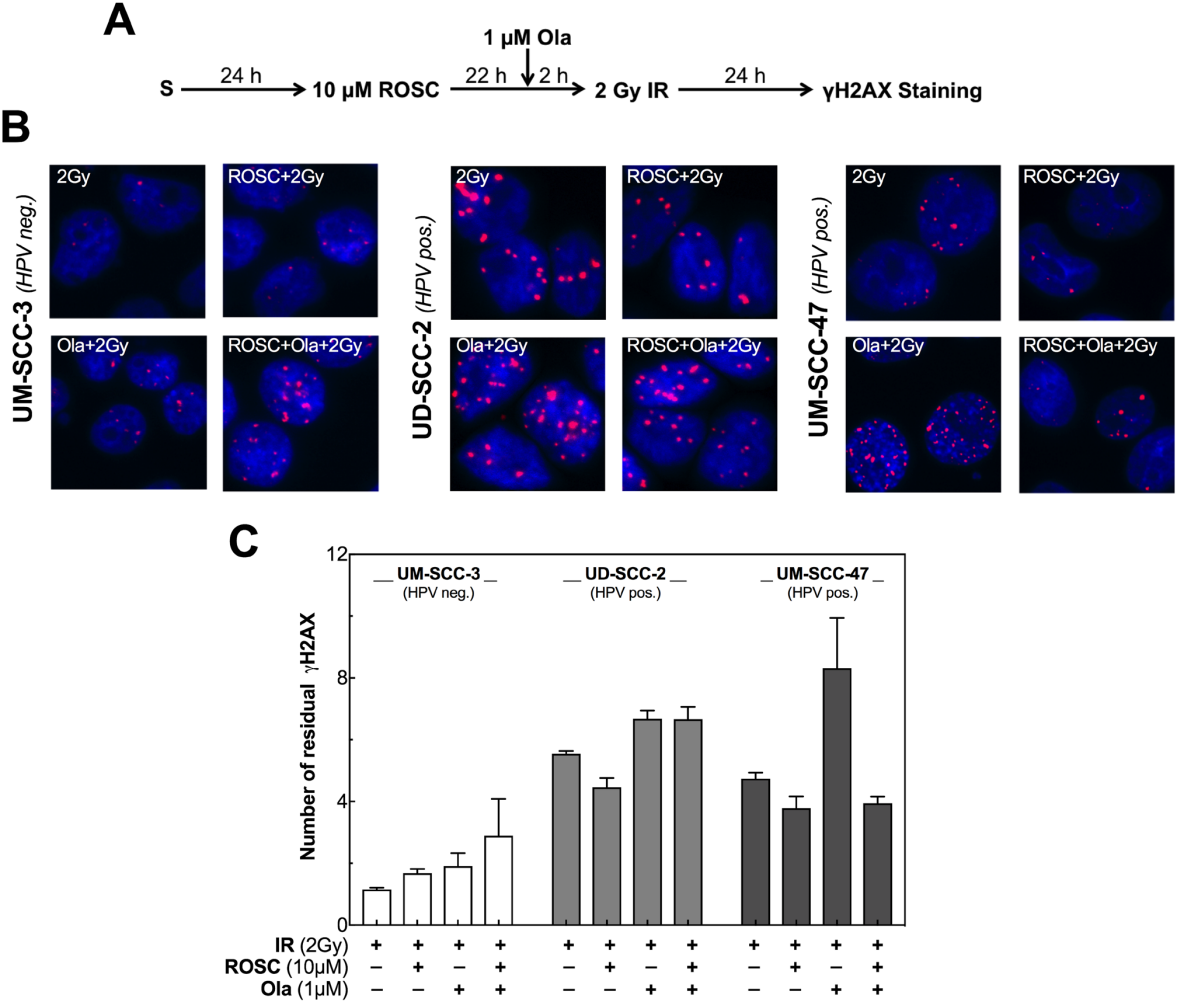
Roscovitine and olaparib affect DSB repair differently in HPV neg. and pos. cell lines **(A)** Scheme of γH2AX staining after ionizing radiation (IR), roscovitine (ROSC) and olaparib (Ola) treatment. **(B)** Representative photos of residual γH2AX foci in UM-SCC-3 (HPV neg., left panel) and UD-SCC-2 as well as UM-SCC-47 cells (HPV pos., middle and right panel) detected 24h after irradiation with 2 Gy alone or after pretreatment either with ROSC (10 μM) or Ola (1 μM) alone or the combination of both. **(C)** Quantification of the number of residual γH2AX foci measured 24 h after IR in UM-SCC-3, UD-SCC-2 as well as UM-SCC-47 cells. The number of foci in the corresponding unirradiated cells was subtracted as background. Quantification was performed by counting at least 200 nuclei per sample. Mean values ± SEM with 3 independent experiments performed per cell line; S = seeding.

We investigated in how far the effect of roscovitine on HR and cell-cycle distribution finally results in an altered number of unrepaired DSBs as can be analyzed by the detection of γH2AX foci. To this end, the number of residual γH2AX foci was measured 24 h after irradiation with 2 Gy using the HPV neg. cell line UM-SCC-3 and the two HPV pos. cell lines UD-SCC-2 and UM-SCC-47 (Figure 4A and 4B). In UM-SCC-3 cells, only few γH2AX foci remained at 24 h after exposure to irradiation with 2 Gy (mean foci: 1.2 ± 0.1). Pretreatment with roscovitine slightly enhanced the number of γH2AX foci 24 h after irradiation (mean foci: 1.7 ± 0.1). In contrast, in the HPV pos. cell lines UD-SCC-2 and UM-SCC-47 clearly more residual DSBs were found 24 h after irradiation alone (mean foci: 5.5 ± 0.1 for UD-SCC-2; 4.7 ± 0.2 for UM-SCC-47), which is in line with the data reported previously [24]. In these cell lines, pretreatment with roscovitine, instead of enhancing the number of residual γH2AX foci, led to a slight reduction of residual DSBs (Figure 4C).

We additionally analyzed the effect of the PARP-inhibitor olaparib in combination with roscovitine on radiation dependent γH2AX Foci. This inhibitor is known to depress the repair of DNA single-strand breaks (SSBs) [22]. Therefore, treatment by olaparib will enhance the number of un-repaired SSBs that collide with replication forks. This leads to one-ended DSBs, which are preferentially repaired by HR. As a consequence, in cells with impaired HR, pretreatment with olaparib will result in an enhanced number of residual DSBs. The data presented above suggest that this DSB repair pathway is impaired in HPV neg. cell lines when pretreated by roscovitine, while HPV pos. cell lines show an intrinsically impaired HR (Figure 2). In agreement with these findings, for the HPV neg. cell line an increase in γH2AX foci was only seen when olaparib was combined with roscovitine, whereas for the two HPV pos. cell lines, the number of residual γH2AX foci was already enhanced when pretreated by olaparib alone. For these cell lines, no further increase, or even a reduction in foci number was seen when cells were additionally pretreated by roscovitine (Figure 4C).

### Roscovitine and olaparib differently affect radiosensitivity in HPV neg. and HPV pos. cell lines

**Figure 5:**
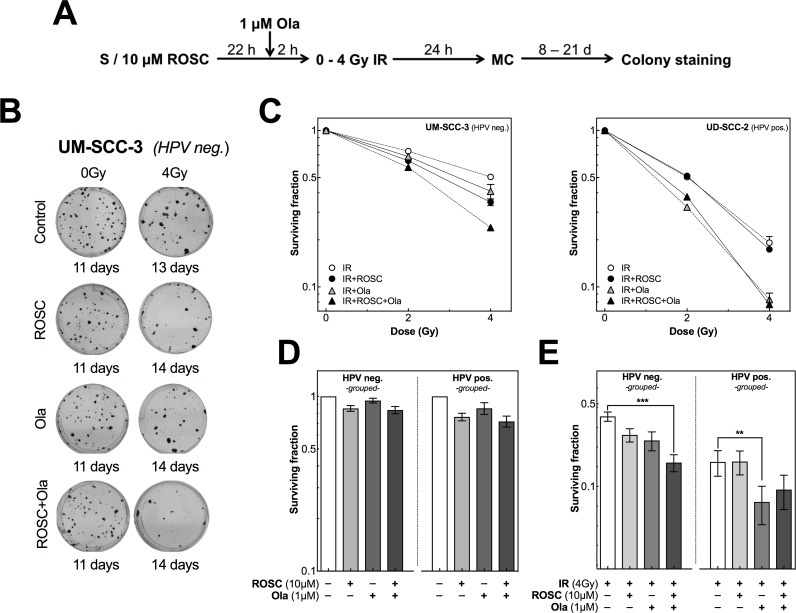
Roscovitine and olaparib affects radiosensitivity differently in HPV neg. and pos. cell lines **(A)** Scheme of colony formation after ionizing radiation (IR), roscovitine (ROSC) and olaparib (Ola) treatment. **(B)** Representative photos of colonies obtained for UM-SCC-3 cells after IR ± treatment with ROSC and/or Ola. Colonies were fixed and stained at different time intervals after IR to allow colonies to grow to equal size. **(C)** Radiosensitivity of UM-SCC-3 (HPV neg., left panel) and UD-SCC-2 (HPV pos., right panel) cells as measured by colony formation assay after pretreatment with ROSC (10 μM) and/or Ola(1 μM) before exposure to IR with the indicated doses. **(D)** Clonogenic survival of HPV neg. (left panel) and HPV pos. (right panel) cells as determined after incubation with ROSC and/or Ola alone. Cells were grouped according to their HPV-status. **(E)** Clonogenic survival of HPV neg. (left panel) and HPV pos. (right panel) cells as measured after 4 Gy alone and after pretreatment with ROSC and/or Ola. Bars represent grouped mean values ± SEM of 5 HPV neg. / 5 HPV pos. cell lines with at least 3 independent experiments performed per cell line; S = seeding; ^*^p<0.05, ^**^p<0.01, ^***^p<0.001.

Finally, we investigated whether the different effects on DSB repair observed for HPV neg. and pos. cell lines treated either with roscovitine or olaparib alone or in combination prior to irradiation will also translate into different effects on cellular survival as determined by colony formation assay (Figure 5A). Both, the HPV neg. cell line UM-SCC-3 as well as the HPV pos. cell line UD-SCC-2 were exposed to irradiation with 0, 2 and 4 Gy either alone or in combination with 10 μM roscovitine and/or 1 μM olaparib (Figure 5B and 5C). To adjust for similar colony growth, cells were stained at different time intervals after irradiation, ranging from 9 up to 21 days. For the HPV neg. cell line both, roscovitine and olaparib, applied as single agent led to a mild increase in radiosensitivity. This effect was clearly enhanced when cells were pretreated with both inhibitors (Figure 5C, left chart). In contrast, for the HPV pos. cell line UD-SCC-2 roscovitine alone did not affect the radiosensitivity at all, while a strong radiosensitization was observed when cells were pretreated by olaparib with no further enhancement when additionally pretreated by roscovitine (Figure 5C, right chart).

Similar data were obtained when colony formation was measured in the other HPV neg. and pos. cell lines using a single dose of 4 Gy (Figure 5D and 5E). Treatment with olaparib alone or in combination with roscovitine affected cell survival to a minor extent in both HPV neg. and pos. cell lines (Figure 5D). When treatment with roscovitine or olaparib was combined with irradiation, generally a small reduction in cell survival was observed for the HPV neg. cell lines, except UT-SCC-33 (Figure 5E; Supplementary Figure 4, left charts). For this cell line, pretreatment with roscovitine did not result in radiosensitization. This corresponds with the observation that HR appears to be slightly impaired in this cell line but not in other HPV neg. cell lines (Supplementary Figure 2). However, when radiation was combined with both roscovitine and olaparib, a significant radiosensitization was seen for all HPV neg. HNSCC cell lines (Figure 5E, Supplementary Figure 4).

As shown for UD-SCC-2 (Figure 5C, right chart) and additionally for the other HPV pos. HNSCC cell lines, no change in radiosensitivity occurred after pretreatment with roscovitine, in contrast to a significant increase in radiosensitivity when cells were pretreated with olaparib (Figure 5E; Supplementary Figure 4, right charts). This effect was largely unaltered when cells were additionally pretreated by roscovitine with, however, some variation. For UM-SCC-104 cells, olaparib alone increased radiosensitivity only to a small extend, which is in agreement with the still partly active HR detected for this particular HPV pos. cell line (Supplementary Figure 2). Surprisingly, for UM-SCC-47 cells the strong radiosensitization achieved by olaparib was even partly diminished when cells were also pretreated by roscovitine.

These data correspond well with our findings on residual γH2AX foci (Figure 4), as also demonstrated by the excellent correlation between the number of residual γH2AX foci measured after the different treatments with the respective SF (Supplementary Figure 5). Overall, these data demonstrate that the effect of roscovitine and olaparib on cellular radiosensitivity clearly depends on the HR capacity of the respective HNSCC cell line and this capacity strongly depends on the HPV status.

## DISCUSSION

The aim of this study was to test, whether the radiosensitivity of HPV neg. and HPV pos. HNSCC cell lines can be enhanced by the CDK inhibitor roscovitine.

The study was performed with HNSCC cell lines previously described to carry the typical features of HPV neg. and pos. HNSCC tumors. For HPV neg. cell lines, these are a low expression of p16, mutated p53 and a low radiosensitivity, while for HPV pos. HNSCC cell lines a high expression of p16, wild-type p53 and an enhanced radiosensitivity are characteristic attributes [24–26]. Previous data also demonstrated that the groups of HPV neg. as well as HPV pos. HNSCC cell lines are characterized by a considerable variation in cellular radio- and chemosensitivity [24–26, 32, 33]. Therefore, experiments were carried out with five cell lines in each group to account for this heterogeneity.

Roscovitine is one of the first generation CDK inhibitors targeting a rather broad spectrum of CDKs via direct binding to the ATP site and with the highest impact on CDK1 and 2. Roscovitine alone was found to cause a slight increase of pCDK1 at T161 and pCDK2 at T160 (Figure 1D–1G), which was considered to result in the moderate G2 arrest detected (Supplementary Figure 3). Roscovitine was also found to suppress cell proliferation in a dose dependent manner with an almost complete block at 20 μM (Figure 1A). At 10 μM, treatment by roscovitine for 48 h also led to a mild reduction of colony formation (Figure 1B). Effects on proliferation and on colony formation appeared to be slightly more pronounced for HPV pos. cell lines. This might be due to the moderate G2 arrest and to the somewhat higher level of apoptosis found for HPV pos. cell lines when treated by roscovitine (Supplementary Figures 3 and 6).

An antiproliferative and cytotoxic effect of roscovitine was already observed in several other tumor models [8–10], which was likewise associated with an enhanced apoptosis as well as G2 arrest as proven here. Gary *et al*. [12], studying the effect of roscovitine on cell viability, also found that HPV pos. HNSCC cell lines possessed a higher sensitivity with, however, a much greater difference between HPV pos. and HPV neg. cell lines than observed in this report.

It was previously reported that DSB repair is generally less effective in HPV pos. cell lines when compared to HPV neg. cell strains [24, 25, 34, 35]. On the basis of RAD51-foci kinetics in CenpF+ cells after irradiation, we show here that all five HPV pos. cell lines are defective in HR, in contrast to an intact HR observed in all five HPV neg. HNSCC cells lines (Figure 2). An impaired HR was previously also detected for the two HPV pos. cell lines UM-SCC-47 and UPCI:SCC154 [35]. Recently, such defect was also proven in a small set of tumor biopsies taken from HPV pos. HNSCC [36]. Overall these data strongly suggest that HPV pos. HNSCC are characterized by a defective HR, which results in a less efficient DSB repair when compared to HPV neg. HNSCC. Certainly, more data obtained from patient derived samples are needed to confirm this important finding.

The underlying molecular mechanisms causing a defect in HR in HPV pos. HNSCC cell lines are not yet fully understood. It was reported that RAD51 is over-expressed in HPV pos. cell lines due to the effect of oncoprotein E7 [34], but that the recruitment of RAD51 to DSBs might be impaired due to a severely reduced expression of the RAD51 loading factor BRCA2 [35]. However, in a large analysis of 89 HNSCC tumor specimens, including 36 HPV pos. samples no such differences or even the opposite trend was found [37]. Recent data indicate that HR might be functionally defective in HPV pos. cell lines, because the up-regulation of p16 causes a down-regulation of cyclin D1, which also implicates on the loading of RAD51 to the sites of DNA damage [38–40].

CDK1 and 2 are also known to be involved in HR. CDKs activate proteins, such as Exo1, BRCA1/2 and CtIp to promote early steps in HR that are necessary to induce RAD51 foci [21–23]. As a consequence, the CDK1/2 inhibitor roscovitine can be used to inhibit HR [41]. This is now also shown here for all five HPV neg. cell lines (Figure 2). In contrast, no effect was achieved in the HPV pos. cell lines, because in these cells HR was already impaired without any treatment (Figure 2). In agreement with this, for HPV neg. but not for HPV pos. cell lines the radiation-induced G2 arrest was strongly enhanced, when cells were pretreated by roscovitine (Figure 3). After combined treatment in HPV neg. cells the G2 arrest was as prominent as in HPV pos. cell lines obtained after irradiation alone (Figure 3B and 3C). These data also imply that the diverging effects seen when HPV neg. and pos. cell lines are treated by roscovitine do not result from a different effect on pCDK1 and pCDK2, since these effects are rather similar (Figure 1), but are primarily caused by the difference in HR competence.

In line with these findings we also observed for HPV neg. cell lines that the radiosensitivity as determined by colony formation assay is clearly enhanced, when cells were pretreated by roscovitine, whereas for HPV pos. cell lines no such radiosensitization occurred (Figure 5). Other authors already reported, that pretreatment by roscovitine may lead to an enhanced radiosensitivity of tumor cells [9, 16, 17]. It is now shown here for HNSCC cell lines for the first time that this effect strongly depends on the HPV status of the cell strains (Figure 5).

A defective HR is generally considered to be an ideal target to achieve a pronounced radiosensitization via the inhibition of PARP1 [42–44]. This inhibition leads to an accumulation of unrepaired SSBs, which - when interfering with replication – may lead to one-ended DSBs, that are specifically repaired by HR. As a consequence, PARP1 inhibition (PARPi) causes lethality in cells deficient in HR. Originally, this concept of synthetic lethality implies a treatment by PARP-inhibition alone, as it is already successfully applied to breast, ovarian and prostate cancer patients defective in HR due to mutations in BRCA1/2 [42, 45]. It was already shown, that this concept may become even more potent when combining PARP-inhibition with irradiation, thereby enhancing the number of unrepaired SSBs probably colliding with replication forks [42–44].

The data presented here indicate, that this extended concept of synthetic lethality should also be applied to HPV pos. and neg. HNSCC tumors. It is confirmed here that the radiosensitivity of HPV pos. HNSCC cell lines can be enhanced by olaparib as already demonstrated by Güster et al. [46] and it is further shown by us, that this is due to a defect in HR. In contrast, for HPV neg. cell lines, which are mostly HR proficient, olaparib alone leads to only a minor radiosensitization (Figure 5). But this effect is strongly enhanced, when HR is depressed by pretreatment with roscovitine. Solely when a HPV neg. cell line is characterized by an impaired HR, an efficient radiosensitization can already be achieved by olaparib alone [47].

The different effects of roscovitine and/or olaparib on HR were found to result in clear differences in residual DSBs finally causing the respective effects measured for the cellular radiosensitivity as indicated by the excellent correlation of these two endpoints in the three cell lines tested (Supplementary Figure 5). This was also true for UM-SCC-47, where surprisingly roscovitine strongly diminished the effect of olaparib in both colony formation and residual DSBs (Supplementary Figure 4, right charts; Figure 5, right chart). This cell line differed from the other HPV pos. cell lines with respect to cell-cycle changes measured after the different treatments (Supplementary Figure 3). We found that solely for this cell line pretreatment by roscovitine led to a decrease of cells in G2 phase with an increase in G1 phase probably suggesting induction of a transient G1 arrest by roscovitine. Due to this increase in G1 phase, UM-SCC-47 cells might have more time to repair DSBs by non-homologous end joining (NHEJ), but most of all also to remove SSBs before entering the S phase even when SSB-repair is depressed by olaparib. As a consequence, less unrepaired SSBs will encounter with replication finally resulting in less residual γH2AX foci leading to an improved cellular survival. After roscovitine treatment, such a protective G1 arrest was also observed by Crescenzi *et al*. [15] for some cancer cell lines treated with doxorubicin.

Overall, our data suggest that the extended concept of synthetic lethality using PARPi and ionizing radiation in HR deficient tumors appears to have a great potential for HNSCC. Due to this concept, HPV pos. tumors with a defective HR should be treated by IR in combination with olaparib alone, while for HPV neg. tumors this combination should be complemented by roscovitine to inhibit HR and thus obtain pharmacologically induced synthetic lethality.

The concept of synthetic lethality always requires a stratification of tumors into HR proficient and deficient ones. However, so far a general and robust marker to identify such a defect in tumor samples is still missing. The data provided here (Figure 2) and especially those obtained for tumor samples by Bhide *et al*. [36] indicate, that – at least for HNSCC – a positive HPV/p16-status may be a robust surrogate for a defective HR.

In summary, our data indicate that the response rates of HPV pos. HNSCC tumors might be further enhanced when combining IR with olaparib and, even more important, that the outcome of patients with HPV neg. tumors might be improved by adding roscovitine to this combination.

## MATERIALS AND METHODS

### Cell lines

The five HPV neg. (UM-SCC-11, UM-SCC-6, UM-SCC-3, UT-SCC-33, UD-SCC-1) and the five HPV pos. (UM-SCC-47, UM-SCC-104, UD-SCC-2, 93-VU147T, UPCI:SCC152) HNSCC cell lines used were described previously by us [25], except UD-SCC-2 (which was provided by H. Bier, Munich, Germany) and UM-SCC-3 (which was provided by T.E. Carey, Michigan USA). Authentication of all cell lines was performed by short tandem repeat analysis at the German Collection of Microorganisms and Cell Cultures (DSMZ, Germany).

### Irradiation (IR) and inhibitors

Irradiation was performed at room temperature using the X-RAD 320 iX X-ray tube (Precision X-Ray Inc.; 320 kV, 10 mA, filter: 0.5 mm Cu + 0.5 mm Al, dose rate: 1.2 Gy/min). For inhibition of CDKs, roscovitine (Calbiochem; Stock 20 mM in DMSO) was added in a final concentration of 10 μM, if not mentioned otherwise. PARP-1 was inhibited by olaparib (Selleckchem; Stock: 10 mM in DMSO) in a final concentration of 1 μM. If not stated otherwise, roscovitine was added 24 h and olaparib 2 h prior to irradiation and removed 24 h later. An equal amount of DMSO was used as solvent control.

### Cell proliferation

For cell proliferation analysis, 8×10^4^ cells were seeded in 3.5 cm petri dishes and counted twice daily for 7 days. Roscovitine was added in increasing doses (0, 5, 10, 20 μM) directly while seeding cells. The number of cells was assed manually using a Neubauer chamber and cell number was calculated relative to the surface area of the petri dish. Dead cells were excluded by trypan blue staining and analyzed separately.

### Colony formation

Cells were seeded in hexaplicates in 6-well plates in various cell numbers, depending on cell line. Roscovitine was added directly to cells while seeding and irradiation was performed 24 h later. In case of PARP-1 inhibition, cells were treated with olaparib 2 h before irradiation. All inhibitors were washed out 24 h after irradiation by a medium change using conditioned medium (fresh medium mixed with cell line specific, filtered supernatant; 3:1). In case of UD-SCC-1, 50% AmnioGrow Plus medium (Cytogen) was added to conditioned medium to facilitate colony formation. For colony formation cells were incubated for 9 to 21 days depending on cell line and treatment. Because treatment with inhibitors or irradiation delayed colony formation, treated samples were allowed to grow for extended periods of time and fixed when colonies reached equal size to control samples. After fixation and staining (10% formaldehyde with 0.1% cristal violet) colonies of at least 50 cells were counted manually and survival was calculated as previously described [32].

### Immunofluorescence

Cells were grown on glass cover slips and incubated with roscovitine 24 h after seeding. After incubation for 24 h cells were irradiated with a dose of 2 Gy. For analysis of RAD51 foci, cells were fixed and stained at various time points (2-24 h) after irradiation with 4% paraform-aldehyde/PBS for 10 min and stored at 4°C over night. For evaluation of γH2AX foci the fixation was performed 24 h after irradiation. Fixed cells were permeabilized with 0.2% Triton X-100, 1% BSA/PBS for 10 min and blocked in 3% BSA/PBS for 1 h. Primary antibody incubation was done for 1 h at room temperature using the following antibodies: mouse monoclonal anti-phospho-S139-H2AX antibody (1:500, clone: JBW301, Millipore), mouse monoclonal anti-RAD51 antibody (1:1000, clone 14b4, Abcam) and rabbit polyclonal anti-CenpF antibody (1:750, Lifespan Bioscience). After washing three times with 0.5% Tween20/PBS for 10 min, the cells were incubated for 1 h with secondary anti-mouse Alexa-fluor594 (1:800, Invitrogen) and anti-rabbit Alexa-fluor488 (1:1200, Invitrogen). Cells were again washed three times and mounted in ProLong Gold antifade reagent (Invitrogen) including DAPI for staining of nuclei. Immunofluorescence was analyzed using the IX81 microscope (objective: 60x, Olympus) and Xcellence Software (Olympus). For analysis z-stacked images were taken from each sample and foci counted manually. The number of foci in irradiated samples was calculated by background subtraction from non-irradiated controls. All experiments were performed at least two times in duplicates and at least 150 nuclei were counted.

### Cell-cycle analysis

For analysis of cell-cycle distribution, cells pretreated with roscovitine (24 h) were fixed at 0, 12, 24 and 48 h after irradiation (6 Gy), stained with propidium iodide and examined by flow cytometry as previously described [32]. The proportion of cells in cell-cycle phases was analyzed using FlowJo V7.6.1 software (Tree Star Inc.).

### Western blotting

Proteins form whole cell extracts were generated and western blot was performed as previously described [32]. The following antibodies from Cell Signaling Technology were used: rabbit anti-phospho-Thr160-CDK1 antibody, rabbit monoclonal anti-CDK2 antibody (clone: 78B2), mouse monoclonal anti-cdc2 antibody (clone: POH1) and rabbit anti-phospho-Thr161-cdc2 antibody all used in a dilution of 1: 1000. Rabbit monoclonal anti-GAPDH antibody (1:3000, clone: 14C10) was used as loading control. Band density was measured using the Quantity One software (Bio-Rad), and values were normalized to corresponding GAPDH loading control.

### Graphs and statistics

If not stated otherwise, all experiments were performed in triplicates and repeated at least three times. Data are presented as mean values ± standard error of the mean (SEM). Students t-test was used to check for statistical significance with significance level p<0.05. All statistical analysis and graphics were made using Graph-Pad Prism 5.0 program (GraphPad Software).

## SUPPLEMENTARY FIGURES



## References

[1] Wittekindt C, Wagner S, Mayer CS, Klussmann JP (2012). Basics of tumor development and importance of human papilloma virus (HPV) for head and neck cancer. GMS Curr Top Otorhinolaryngol Head Neck Surg.

[2] Simard EP, Torre LA, Jemal A (2014). International trends in head and neck cancer incidence rates: Differences by country, sex and anatomic site. Oral Oncol.

[3] Nguyen-Tan PF, Zhang Q, Ang KK, Weber RS, Rosenthal DI, Soulieres D, Kim H, Silverman C, Raben A, Galloway TJ, Fortin A, Gore E, Westra WH (2014). Randomized phase III trial to test accelerated versus standard fractionation in combination with concurrent cisplatin for head and neck carcinomas in the radiation therapy oncology group 0129 trial: Long-term report of efficacy and toxicity. J Clin Oncol.

[4] Möckelmann N, Kriegs M, Lörincz BB, Busch CJ, Knecht R (2016). Molecular targeting in combination with platinum-based chemoradiotherapy in head and neck cancer treatment. Head Neck.

[5] Martins RG, Parvathaneni U, Bauman JE, Sharma AK, Raez LE, Papagikos MA, Yunus F, Kurland BF, Eaton KD, Liao JJ, Mendez E, Futran N, Wang DX (2013). Cisplatin and radiotherapy with or without erlotinib in locally advanced squamous cell carcinoma of the head and neck: A randomized phase II trial. J Clin Oncol.

[6] Lapenna S, Giordano A (2009). Cell cycle kinases as therapeutic targets for cancer. Nat Rev Drug Discov.

[7] Johnson N, Shapiro GI (2010). Cyclin-dependent kinases (cdks) and the DNA damage response: rationale for cdk inhibitor-chemotherapy combinations as an anticancer strategy for solid tumors. Expert Opin Ther Targets.

[8] Cicenas J, Kalyan K, Sorokinas A, Stankunas E, Levy J, Meskinyte I, Stankevicius V, Kaupinis A, Valius M (2015). Roscovitine in cancer and other diseases. Ann Transl Med.

[9] Hui ABY, Yue S, Shi W, Alajez NM, Ito E, Green SR, Frame S, O'Sullivan B, Liu FF (2009). Therapeutic efficacy of seliciclib in combination with ionizing radiation for human nasopharyngeal carcinoma. Clin Cancer Res.

[10] Tirado OM, Mateo-Lozano S, Notario V (2005). Roscovitine is an effective inducer of apoptosis of Ewing's sarcoma family tumor cells *in vitro* and *in vivo*. Cancer Res.

[11] Mihara M, Shintani S, Kiyota A, Matsumura T, Wong DTW (2002). Cyclin-dependent kinase inhibitor (roscovitine) suppresses growth and induces apoptosis by regulating Bcl-x in head and neck squamous cell carcinoma cells. Int J Oncol.

[12] Gary C, Hajek M, Biktasova A, Bellinger G, Yarbrough WG, Issaeva N (2016). Selective antitumor activity of roscovitine in head and neck cancer. Oncotarget.

[13] Benson C, White J, De Bono J, O'Donnell A, Raynaud F, Cruickshank C, McGrath H, Walton M, Workman P, Kaye S, Cassidy J, Gianella-Borradori A, Judson I (2007). A phase I trial of the selective oral cyclin-dependent kinase inhibitor seliciclib (CYC202; R-Roscovitine), administered twice daily for 7 days every 21 days. Br J Cancer.

[14] Le Tourneau C, Faivre S, Laurence V, Delbaldo C, Vera K, Girre V, Chiao J, Armour S, Frame S, Green SR, Gianella-Borradori A, Diéras V, Raymond E (2010). Phase I evaluation of seliciclib (R-roscovitine), a novel oral cyclin-dependent kinase inhibitor, in patients with advanced malignancies. Eur J Cancer.

[15] Crescenzi E, Palumbo G, Brady HJM (2005). Roscovitine modulates DNA repair and senescence: Implications for combination chemotherapy. Clin Cancer Res.

[16] Maggiorella L, Deutsch E, Frascogna V, Maggiorella L, Deutsch E, Chavaudra N, Jeanson L, Milliat F (2003). Enhancement of radiation response by roscovitine in human breast carcinoma *in vitro* and *in vivo*. Cancer Res.

[17] Zhang F, Zhang T, Gu ZP, Zhou YA, Han Y, Li XF, Wang XP, Cheng QS, Mei QB (2008). Enhancement of radiosensitivity by roscovitine pretreatment in human non-small cell lung cancer A549 cells. J Radiat Res.

[18] Korinkova G, Cwiertka K, Paprskarova M, Dzubak P, Hajduch M (2010). The radiosensitising effect of olomoucine derived synthetic cyclin-dependent kinase inhibitors. Neoplasma.

[19] Kasten-Pisula U, Tastan H, Dikomey E (2005). Huge differences in cellular radiosensitivity due to only very small variations in double-strand break repair capacity. Int J Radiat Biol.

[20] Mansour WY, Schumacher S, Rosskopf R, Rhein T, Schmidt-Petersen F, Gatzemeier F, Haag F, Borgmann K, Willers H, Dahm-Daphi J (2008). Hierarchy of nonhomologous end-joining, single-strand annealing and gene conversion at site-directed DNA double-strand breaks. Nucleic Acids Res.

[21] Tomimatsu N, Mukherjee B, Catherine Hardebeck M, Ilcheva M, Vanessa Camacho C, Louise Harris J, Porteus M, Llorente B, Khanna KK, Burma S (2014). Phosphorylation of EXO1 by CDKs 1 and 2 regulates DNA end resection and repair pathway choice. Nat Commun.

[22] Johnson N, Li YC, Walton ZE, Cheng KA, Li D, Rodig SJ, Moreau LA, Unitt C, Bronson RT, Thomas HD, Newell DR, D'Andrea AD, Curtin NJ (2011). Compromised CDK1 activity sensitizes BRCA-proficient cancers to PARP inhibition. Nat Med.

[23] Wang H, Shi LZ, Wong CCL, Han X, Hwang PY, Truong LN, Zhu Q, Shao Z, Chen DJ, Berns MW, Yates JR, Chen L, Wu X (2013). The interaction of CtIP and Nbs1 connects CDK and ATM to regulate HR–mediated double-srand break repair. PLoS Genet.

[24] Rieckmann T, Tribius S, Grob TJ, Meyer F, Busch CJ, Petersen C, Dikomey E, Kriegs M (2017). HNSCC cell lines positive for HPV and p16 possess higher cellular radiosensitivity due to an impaired DSB repair capacity. Radiother Oncol.

[25] Arenz A, Ziemann F, Mayer C, Wittig A, Dreffke K, Preising S, Wagner S, Klussmann JP, Engenhart-Cabillic R, Wittekindt C (2014). Increased radiosensitivity of HPV-positive head and neck cancer cell lines due to cell cycle dysregulation and induction of apoptosis. Strahlenther Onkol.

[26] Kimple R, Smith M, Blitzer G, Torres A (2013). Enhanced radiation sensitivity in HPV-positive head and neck cancer. Cancer Res.

[27] Coulonval K, Kooken H, Roger PP (2011). Coupling of T161 and T14 phosphorylations protects cyclin B-CDK1 from premature activation. Mol Biol Cell.

[28] Deckbar D, Jeggo PA, Löbrich M (2011). Understanding the limitations of radiation-induced cell cycle checkpoints. Crit Rev Biochem Mol Biol.

[29] Trovesi C, Manfrini N, Falcettoni M, Longhese MP (2013). Regulation of the DNA damage response by cyclin-dependent kinases. J Mol Biol.

[30] Beucher A, Birraux J, Tchouandong L, Barton O, Shibata A, Conrad S, Goodarzi AA, Krempler A, Jeggo PA, Löbrich M (2009). ATM and Artemis promote homologous recombination of radiation-induced DNA double-strand breaks in G2. EMBO J.

[31] Branzei D, Foiani M (2008). Regulation of DNA repair throughout the cell cycle. Nat Rev Mol Cell Biol.

[32] Ziemann F, Arenz A, Preising S, Wittekindt C, Klussmann JP, Engenhart-Cabillic R, Wittig A (2015). Increased sensitivity of HPV-positive head and neck cancer cell lines to x-irradiation +/− Cisplatin due to decreased expression of E6 and E7 oncoproteins and enhanced apoptosis. Am J Cancer Res.

[33] Busch CJ, Becker B, Kriegs M, Gatzemeier F, Krüger K, Möckelmann N, Fritz G, Petersen C, Knecht R, Rothkamm K, Rieckmann T (2016). Similar cisplatin sensitivity of HPV-positive and -negative HNSCC cell lines. Oncotarget.

[34] Park JW, Nickel KP, Torres AD, Lee D, Lambert PF, Kimple RJ (2014). Human papillomavirus type 16 E7 oncoprotein causes a delay in repair of DNA damage. Radiother Oncol.

[35] Weaver AN, Cooper TS, Rodriguez M, Trummell HQ, Bonner JA, Rosenthal EL, Yang ES (2015). DNA double strand break repair defect and sensitivity to poly ADP-ribose polymerase (PARP) inhibition in human papillomavirus 16-positive head and neck squamous cell carcinoma. Oncotarget.

[36] Bhide SA, Thway K, Lee J, Wong K, Clarke P, Newbold KL, Nutting CM, Harrington KJ (2016). Delayed DNA double-strand break repair following platin-based chemotherapy predicts treatment response in head and neck squamous cell carcinoma. Br J Cancer.

[37] Moeller BJ, Yordy JS, Williams MD, Giri U, Raju U, Molkentine DP, Byers LA, Heymach JV, Story MD, Lee JJ, Sturgis EM, Weber RS, Garden AS (2011). DNA repair biomarker profiling of head and neck cancer: Ku80 expression predicts locoregional failure and death following radiotherapy. Clin Cancer Res.

[38] Dok RR, Kalev P, Van Limbergen EJ, Asbagh LA, Vázquez I, Hauben E, Sablina A, Nuyts S, Vazquez I, Hauben E, Sablina A, Nuyts S (2014). P16INK4a impairs homologous recombination-mediated DNA repair in human papillomavirus-positive head and neck tumors. Cancer Res.

[39] Dok R, Abbasi Asbagh L, Van Limbergen EJ, Sablina A, Nuyts S (2016). Nuclear p16INK4a expression predicts enhanced radiation response in head and neck cancers. Oncotarget.

[40] Chalermrujinanant C, Michowski W, Sittithumcharee G, Esashi F, Jirawatnotai S (2016). Cyclin D1 promotes BRCA2-Rad51 interaction by restricting cyclin A/B-dependent BRCA2 phosphorylation. Oncogene.

[41] Mansour WY, Borgmann K, Petersen C, Dikomey E, Dahm-Daphi J (2013). The absence of Ku but not defects in classical non-homologous end-joining is required to trigger PARP1-dependent end-joining. DNA Repair (Amst).

[42] Helleday T (2011). The underlying mechanism for the PARP and BRCA synthetic lethality: Clearing up the misunderstandings. Mol Oncol.

[43] Kötter A, Cornils K, Borgmann K, Dahm-Daphi J, Petersen C, Dikomey E, Mansour WY (2014). Inhibition of PARP1-dependent end-joining contributes to Olaparib-mediated radiosensitization in tumor cells. Mol Oncol.

[44] Powell C, Mikropoulos C, Kaye SB, Nutting CM, Bhide SA, Newbold K, Harrington KJ (2010). Pre-clinical and clinical evaluation of PARP inhibitors as tumour-specific radiosensitisers. Cancer Treat Rev.

[45] Benafif S, Hall M (2009). An update on PARP inhibitors for the treatment of cancer. Onco Targets Ther.

[46] Guster JD, Weissleder SV, Busch CJ, Kriegs M, Petersen C, Knecht R, Dikomey E, Rieckmann T (2014). The inhibition of PARP but not EGFR results in the radiosensitization of HPV/p16-positive HNSCC cell lines. Radiother Oncol.

[47] Wurster S, Hennes F, Parplys AC, Seelbach JI, Mansour WY, Zielinski A, Petersen C, Clauditz TS, Münscher A, Friedl AA, Borgmann K (2016). PARP1 inhibition radiosensitizes HNSCC cells deficient in homologous recombination by disabling the DNA replication fork elongation response. Oncotarget.

