# Supplementary material for: Roscovitine strongly enhances the effect of olaparib on radiosensitivity for HPV neg. but not for HPV pos. HNSCC cell lines
Source: Oncotarget. 2017 Oct 24;8(62):105170–83. doi: 10.18632/oncotarget.22005 (PMC5739629; doi:10.18632/oncotarget.22005)
Supplement: Supplementary file 1 [file oncotarget-08-105170-s001.pdf]

Roscovitine strongly enhances the effect of olaparib on radiosensitivity for HPV neg. but not for HPV pos. HNSCC cell lines

SUPPLEMENTARY MATERIALS

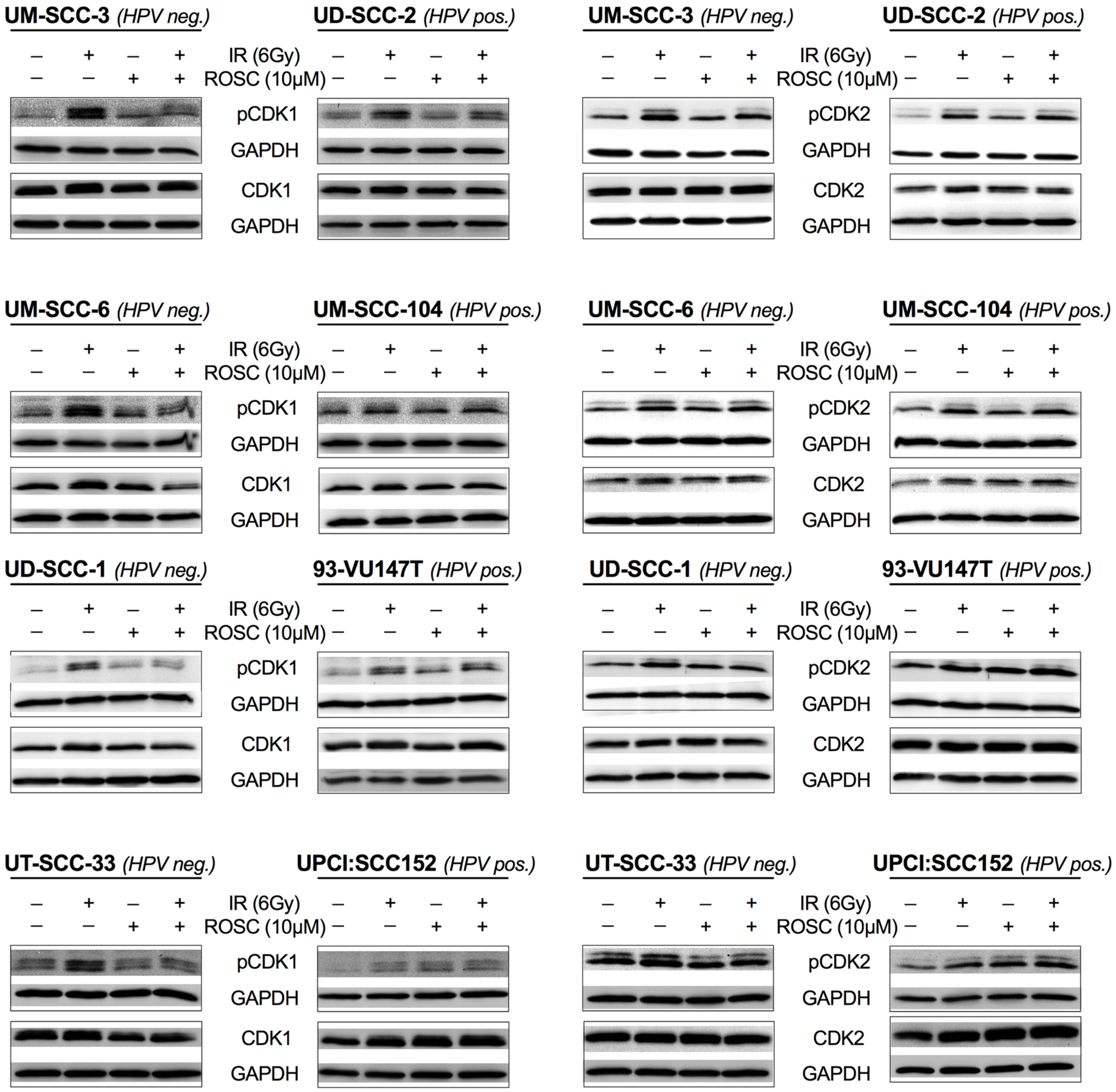

Supplementary Figure 1: Activating phosphorylation and total protein level of CDK1 (left panel) and CDK2 (right panel) 12 h after radiation (6 Gy) and/or roscovitine (10 μM) pretreatment. Shown are representative Western blots for each individual HPV neg. (far left and middle right panel) and HPV pos. (middle left and far right panel) cell line. Radiation increased phosphorylation of CDK1 and CDK2, while roscovitine suppressed this increase, indicating less activating phosphorylation of CDKs.

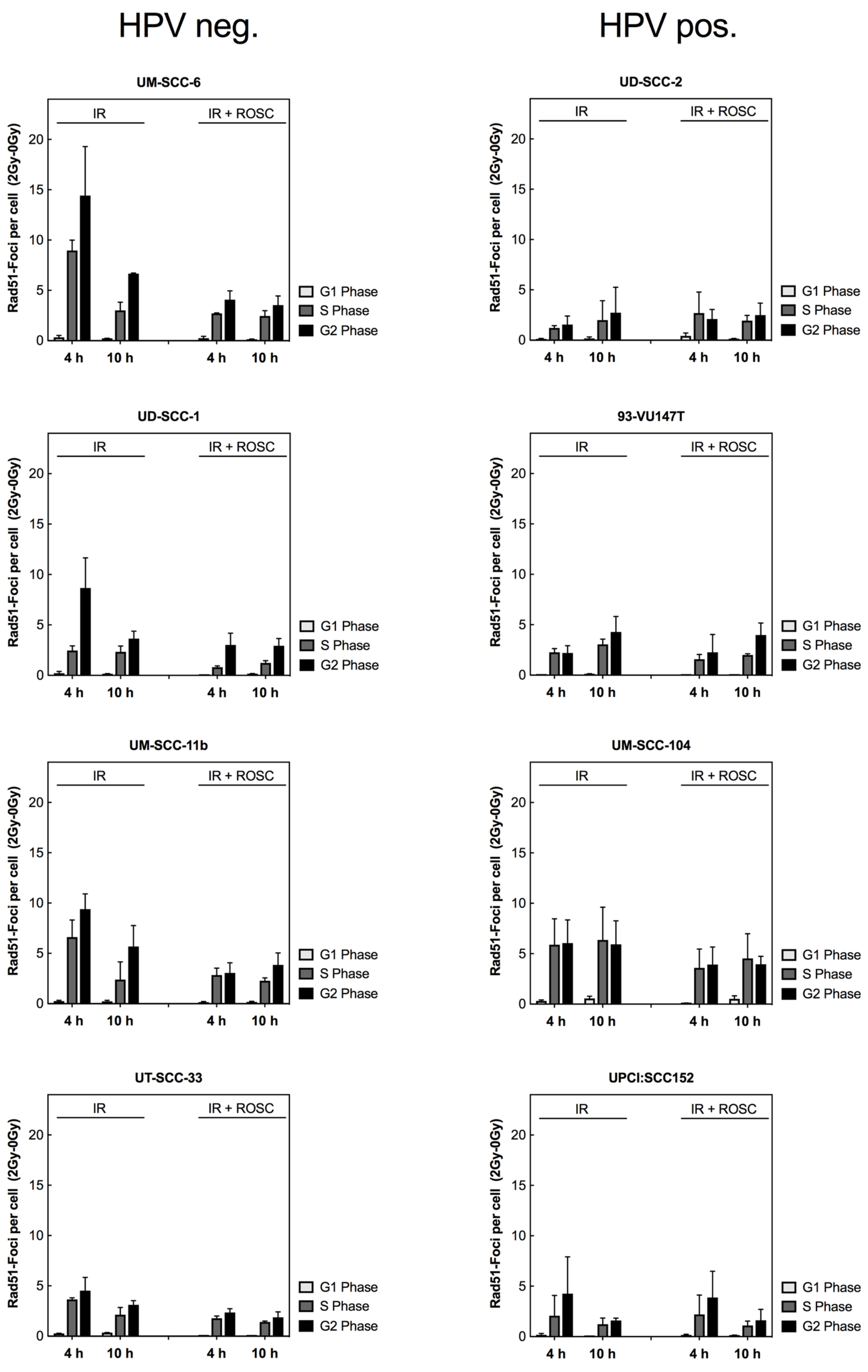

Supplementary Figure 2: Numbers of RAD51 foci in HPV neg. (left panel) and HPV pos. (right panel) HNSCC cell lines in different cell-cycle phases (G1, S, G2) determined by CenpF staining. Data shows the number of foci 4 and 10 h after exposure to 2 Gy (IR) alone or after a pretreatment with roscovitine (ROSC) for each cell line tested. Values are corrected for the number of foci detected in unirradiated samples. Mean values ± SEM.

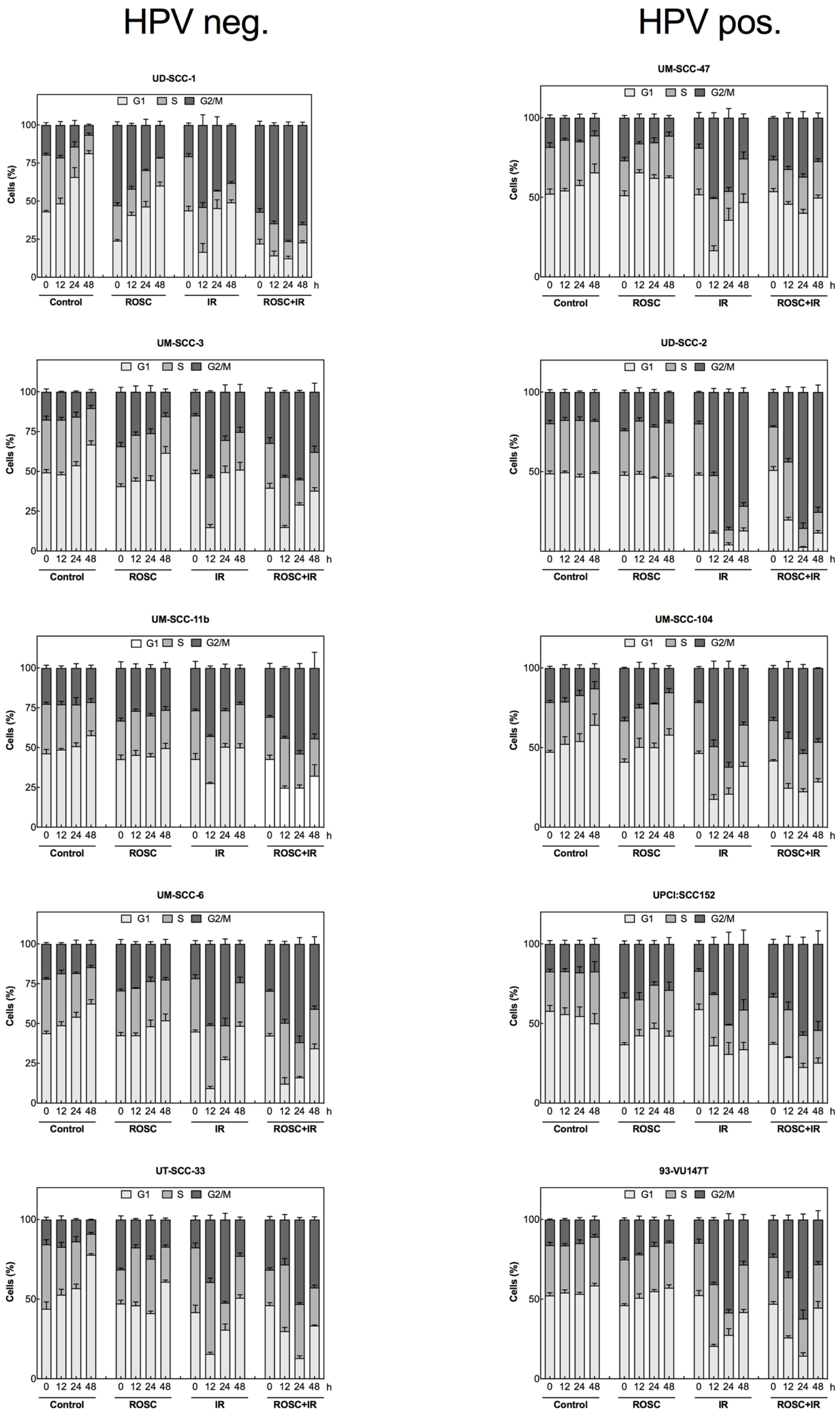

Supplementary Figure 3: Cell-cycle distribution determined for each HPV neg. (left panel) and each HPV pos. (right panel) HNSCC cell line. Stacked bars show percentage of cells in G1 (white), S (light gray) and G2 (dark grey) phase 0, 12, 24 and 48 h after radiation (IR). In case of roscovitine (ROSC) treatment cells were incubated with 10 µM ROSC 24 h prior to IR. Bars are grouped in treatment groups with control, ROSC, IR, ROSC+IR (from left to right). Mean values ± SEM.

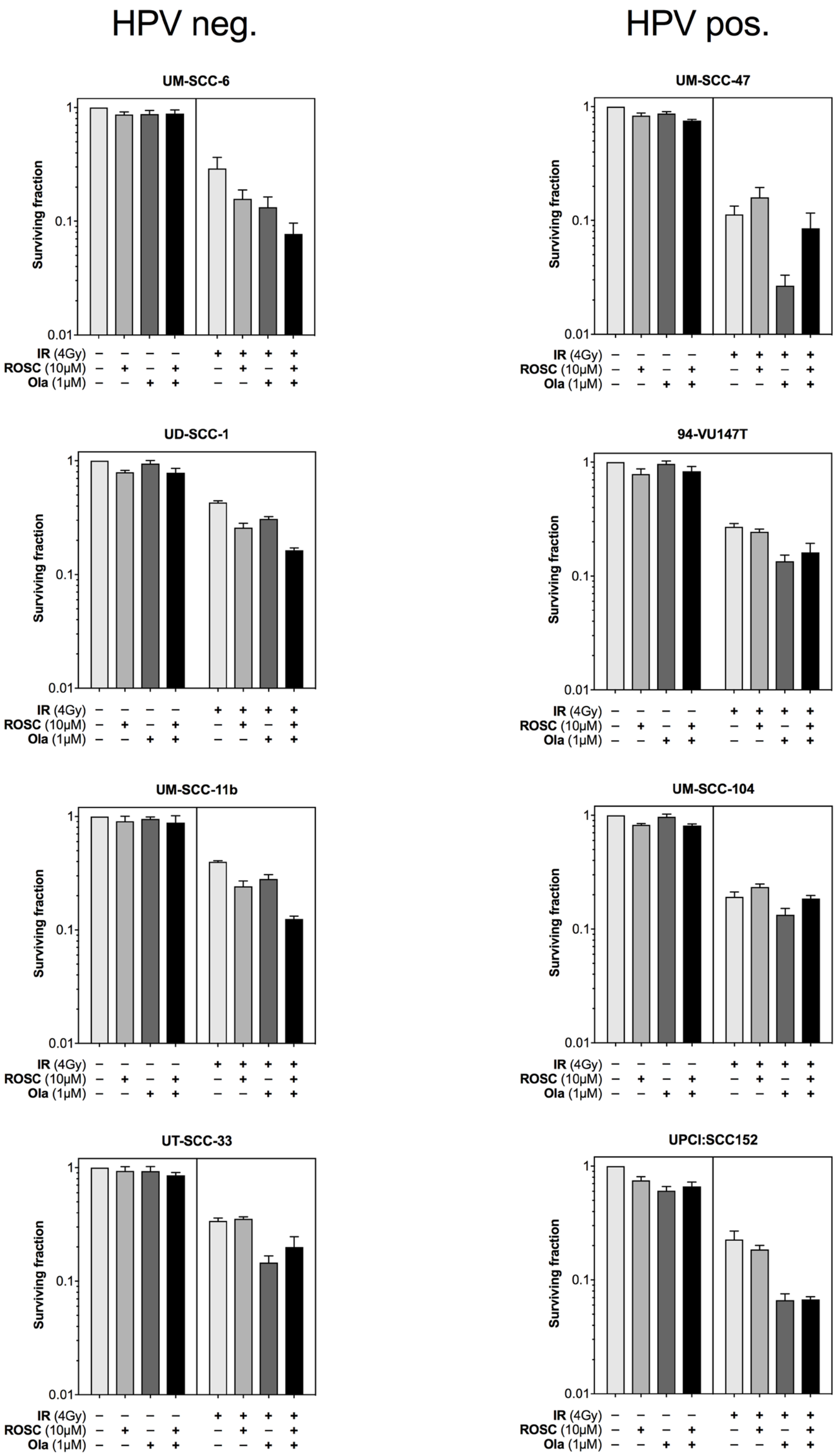

Supplementary Figure 4: Clonogenic survival determined for each HPV neg. (left panel) or HPV pos. (right panel) HNSCC cell line. Cell lines were either treated with radiation (IR, 4 Gy), roscovitine (ROSC, 10 µM), or olaparib (Ola, 1 µM) alone or by the combination of two (ROSC+Ola, ROSC+IR, Ola+IR) or all three treatments (ROSC+Ola+IR). After irradiation cells were incubated for 24 h before medium was changed and colonies were allowed to grow until equal size was achieved. After treatment with roscovitine or olaparib alone surviving fraction was corrected for the plating efficiency; irradiated samples were additionally corrected for the effects of the inhibitors alone. Mean values ± SEM.

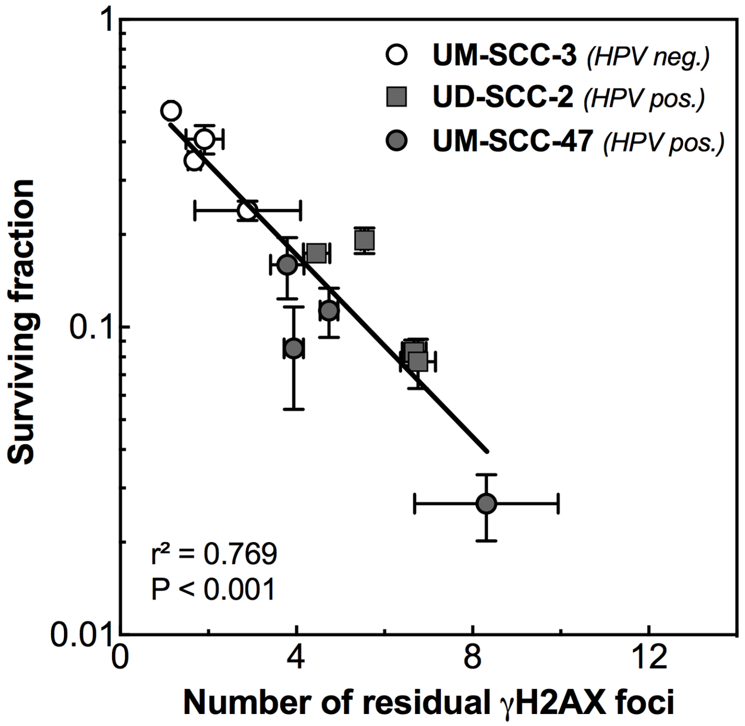

Supplementary Figure 5: Association between the surviving fraction (SF) and the number of residual γH2AX foci measured for UM-SCC-3 (HPV neg.), UD-SCC-2 and UM-SCC-47 (HPV pos.) cell lines after irradiation alone or in combination with roscovitine and/or olaparib. For cell survival, data were taken from Figure 5 and supplement Figure S4 and for residual γH2AX foci from Figure 4. Data could be described by one single correlation demonstrating that independent of the treatment used a change in survival is associated with a respective variation in the number of residual γH2AX foci. These data demonstrates that the change in survival measured after the different treatments are mainly achieved by an interference with DSB repair capacity. Mean values ± SEM.

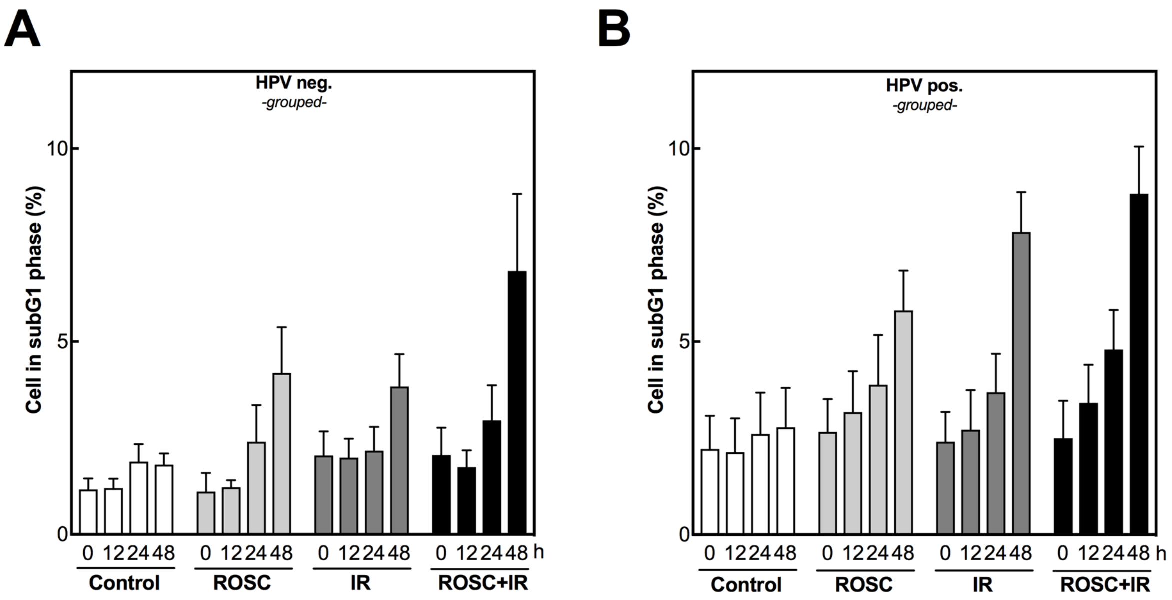

Supplementary Figure 6: Higher amount of apoptosis in HPV pos. cells after irradiation, roscovitine alone or a combined treatment when compared to HPV neg. HNSCC cell lines. Fraction of cells in the subG1 as determined from cell-cycle analysis 0, 12, 24 or 48 h after radiation (IR, 6 Gy) and or roscovitine (ROSC, 10 µM). Roscovitine was administered 24 h prior to IR and not removed during the period of analysis. Shown is the grouped amount of cells in subG1 fraction for all five HPV neg. **(A)** and all five HPV pos. **(B)** cell lines. At all time points and in all treatment groups only a small amount of dead cells (<10 %) is observed which was slightly higher for HPV pos. cell lines, indicating that apoptotic cell death is not the primary event during the first 48 h after ROSC±IR in HNSCC cell lines. Bars represent grouped mean values ± SEM of 5 HPV neg. / 5 HPV pos. cell lines.
